# Combined Effect of Acid Whey Addition and Ultrasonic Treatment on the Chemical and Microbiological Stability of Lamb Stuffing

**DOI:** 10.3390/foods12071379

**Published:** 2023-03-24

**Authors:** Agnieszka Latoch, Dariusz M. Stasiak, Andrzej Junkuszew

**Affiliations:** 1 Department of Animal Food Technology, University of Life Sciences in Lublin, 20-400 Lublin, Poland; 2 Department of Animal Breeding and Agricultural Consulting, University of Life Sciences in Lublin, 20-400 Lublin, Poland

**Keywords:** lamb, byproduct of cheesemaking, ultrasound, technological properties, stuffing

## Abstract

The microbiological and chemical stability of stuffing is crucial in meat processing. Small ruminant (lamb) meat has many nutritional advantages (fatty acid composition and ratio, high biological value of protein, source of zinc, and selenium) but is poorly utilized in processing. In this experiment, we determined the effects of physical (sonication), chemical (salt, curing salt, and air access), and microbiological (acid whey) factors on the microbiological and chemical stability of lamb meat stuffing. Proximate composition and fatty acids profile, pH, water activity, lipid oxidation, color parameters, and microbiology examinations were performed in fresh meat stuffing and on the fifth day of refrigerated storage. Ultrasound treatment of the meat with whey reduced its acidity and increased the oxidative stability of fats but did not modify the water activity and color of the stuffing. Stuffing sonication did not affect the growth of LAB but reduced the number of *Entereobacteriace*, especially in the presence of whey and salt. The treatment of lamb meat stuffing with low-frequency and medium-intensity ultrasound assisted by the addition of acid whey and salt is a technique conducive to reducing the use of nitrates in meat technology and their consumption by consumers.

## 1. Introduction

Food safety is a serious challenge for science and the industry regardless of the part of the world. A large proportion of consumers are aware of what they eat and their requirements for quality food. Lamb meat, compared to poultry, pork, or beef, makes up a relatively small portion of the human diet [1], even though it contains many important nutrients, such as essential amino acids, vitamins (e.g., niacin, pantothenic acid, and cobalamin), macro- and micronutrients such as potassium, iron, selenium, and phosphorus [2,3]. Consuming lamb meat, especially from native breeds, can provide a few dietary benefits but also sustainability and food security benefits.

Minced meat is commonly used in meat technology as a sausage stuffing or an ingredient in meat dishes. However, minced meat is one of the most microbiologically labile food products due to its fineness and chemical composition. Favorable conditions for microbial growth and oxidation reactions, numerous enzymes, prooxidants, and especially large surface areas accelerate oxidative changes and microbial growth [4]. Lipid oxidation is one of the main phenomena of non-microbial spoilage of mutton meat due to the high proportion of unsaturated fatty acids (45% monounsaturated and 10% polyunsaturated ones) in the total pool of fatty acids. Microbial growth is the main cause of meat deterioration during cold storage [4]. Both spoilage and microbial growth occur during meat storage, causing consumer disapproval, economic losses, and serious health risks for consumers. Minced meat usually spoils faster than unground meat. However, the introduction of new and modification of existing processing techniques through the results of scientific research can improve the quality and stability of processed meat.

Elimination of pathogens present in meat and meat products is crucial to food safety. One common method of eliminating pathogens is the addition of nitrite. Nitrite in meat technology is a multifunctional additive: it perpetuates the pink-red color, provides oxidative and microbiological stabilization during storage, and improves sensory properties. However, due to concerns over the excessive use of nitrate in meat products [5,6] researchers are looking for strategies to reduce its use [7]. Most nitrate-free curing alternatives provide only one effect, e.g., obtaining and stabilizing color, antioxidant activity, or bactericidal activity only. Therefore, the best way to use these substances is to combine several independent factors (antioxidant, color-forming, and bacteriostatic) to achieve the desired effect comparable to the use of nitrates [6]. Based on the research conducted so far [8,9] it has been found that achieving the desired sensory characteristics and adequate microbiological stability can be achieved by using acid whey. The low pH of whey and the lactic acid bacteria present in it show great potential to counteract the growth of pathogenic microorganisms and favorably affect the stabilization of the quality of meat products. Previous research on the use of acid whey as a substitute for nitrates has mainly focused on adding it in liquid form to the production of raw maturing meat products [8,9]. There are no studies in the literature on the effect of adding liquid acid whey to lamb meat stuffing, stored for several days under a vacuum, on the microbiological and chemical stability.

The use of acid whey in the production of nitrate-free meat products in industrial practice can be difficult, especially due to the different microbiological quality of traditionally obtained acid whey [10]. Therefore, it is necessary to implement additional microbiological barriers, preferably acting synergistically, to ensure the health and safety of unpasteurized intermediate products. This can be achieved using an optimized combination of several factors, such as specific physical interaction, lowering the pH, increasing the concentration of sodium chloride, properly managing a refrigerated supply chain, and reducing storage time. A low-frequency and medium-intensity ultrasound technique demonstrates quite high application potential.

Ultrasonic technology is a constantly developing scientific sphere with a high potential for technological innovation. The impact of ultrasound is physical in nature and involves the absorption of mechanical waves in the environment. The nature of the impact depends primarily on the acoustic properties of the environment, the frequency of the waves, and their intensity. The optimal conditions for the interaction of ultrasound in biological media are provided by low frequency (in the range of 20–60 kHz) and medium intensity. Then, a wide spectrum of physical, chemical, and microbiological impacts of direct and secondary nature is obtained, but with limited dissipation effects [11,12,13]. The mechanism of microbial inhibition involves the occurrence of ultrasonic cavitation. Implosions of cavitation bubbles induce tremendous mechanical stresses and local temperature rise, causing the destruction of cell membranes. Free radicals, on the other hand, exhibit antimicrobial activity by, among others, damaging DNA. The effects of sonication, especially antimicrobial efficacy, are determined by factors relating to the wave source (frequency and intensity), material (physical and chemical properties and biological structure), and technique (duration of treatment, pressure, temperature, and presence of ions) [14,15,16]. Ultrasound-assisted food processing is considered a safe, non-chemical method. It enhances its safety while minimizing nutritional value and sensory quality deterioration compared to, for example, heat treatment by traditional methods. In addition, by combining the action of ultrasound with other techniques (e.g., thermosonication, mansonication, chemosonication), higher antimicrobial efficiency can be achieved. The complexity of the interaction of ultrasound waves and biological structures (e.g., biological tissues and cells) usually requires empirical optimization of process parameters, both on a laboratory and industrial scale [17].

Based on the available literature data, the hypothesis was made that ultrasonic treatment of lamb meat combined with the action of acid whey components would provide the required shelf life and safety of lamb stuffing for further industrial processing, catering, and self-preparation by the final consumer. Accordingly, the necessary range of laboratory tests was determined, which included: pH, fat oxidation, water activity, color parameters, and growth of selected bacteria (total viable count (TVC), lactic acid bacteria count (LAB), *Enterobacteriaceae*, *Escherichia coli*, *Listeria monocytogenes*, and *Clostridium perfringens*). This study was conducted by comparing the chemical and microbiological properties of different lamb stuffing variants prepared with added acid whey and the use of ultrasonic treatment. The type of interaction between the factors and the possible technological advantages in processing lamb without chemical additives were sought.

## 2. Materials and Methods

### 2.1. Preparation of Meat

The meat for this study originated from lambs of the native Polish Wrzosówka breed of sheep from a farm run on organic principles in the Lublin region of Poland. The primary feed during the 1st rearing period (3 months) was milk from the mothers. Feeding of lambs with solid feed (meadow hay and crushed oat grain) was carried out from the second week of life. From the 5th week on, lambs received juicy roughage in the form of hydrated pulp. Lambs, in the first period of rearing, had free access to feed, licks (cubed salt), and water. The lambs were separated from their mothers after 90 days. From then on, the basis of the feed ration was meadow hay (ad libitum), a mixture of grass and alfalfa (limited), and crushed oat grain (limited). The feed ration was adjusted every two weeks according to the animals’ development. Four-month-old lambs were transferred to xerothermic grasslands (Natura 2000), where further fattening was carried out. The body weight of the rams at birth was about 3 kg, after 28 days—7.7 kg, and after 56 days—14.1 kg. Animals at the age of 7–8 months weighing about 27 kg were selected from the herd, marked and taken over by a commercial plant conducting industrial slaughter of animals under the supervision of the State Veterinary Inspection. This plant is registered and approved by state supervision authorities, and processes are carried out in compliance with strict EU regulations on animal welfare, hygiene of operations, and product quality. Fresh meat from the cutting of lamb carcasses was purchased from the plant on a commercial basis and transported to the laboratory. The slaughter yield achieved was 43.1%.

### 2.2. Preparation of Acid Whey

Fresh acid whey was achieved from a local SME dairy from cottage cheese production. The fresh unpasteurized milk was acidified with natural microflora (sourdough) and heated to 40 °C. The whey was separated from the curds in the next step. The number of lactic acid bacteria (LAB) in the acid whey was 4.15 log CFU ml^−1^. The test was performed in accordance with ISO 15214:1998 [18].

### 2.3. Model Meat Stuffing

**Figure 1:**
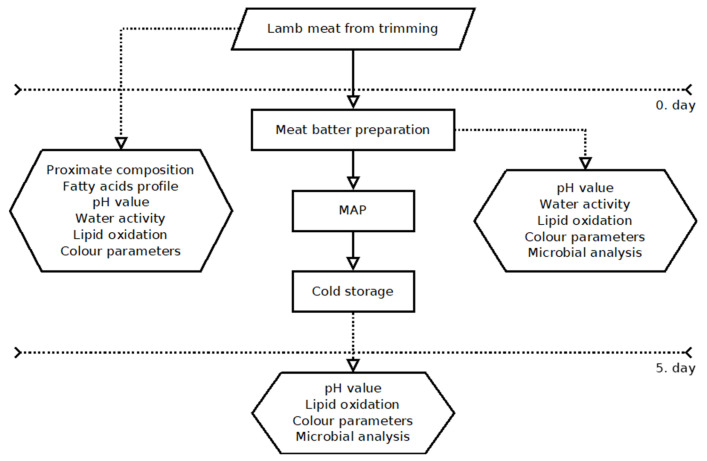
Stuffing preparation and testing scheme.

The model meat stuffing was prepared by mincing fresh (24 h after slaughtering) fine lamb meat, using a laboratory wolf, equipped with a mesh of 12 mm in diameter. The stuffing, after adding variant-specific ingredients (Table 1), was mixed using a KU2-3EK type universal machine (MESKO-AGD, Skarżysko-Kamienna, Poland) until a plastic compact mass was obtained. The stuffing was sonicated in a laboratory device equipped with an electric generator and piezoelectric transducers generating waves with a fundamental frequency of 28 kHz and an intensity of about 2 W cm^−2^. The transducer (of the sandwich type) was fixed under the bottom of a working tank made of acid-resistant sheet metal 0.7 mm thick. A portion of the stuffing in the shape of a cylinder with a diameter of 70 mm was placed directly above the transducer on the water-wetted bottom of the working chamber. The duration of the procedure was 150 s. Sonication parameters were selected based on previous experiments [19]. Ten variants of stuffing were prepared (Table 1). Portions of 400 g of stuffing were vacuum-packed in transparent bags and placed at 4 °C. The stuffing was tested directly after preparation (0 days) and then after 5 days of storage, according to Figure 1.

### 2.4. Proximate Composition of Meat

Samples were analyzed with a near-infrared spectrometer FoodScan™ (FOSS Analytical, Hillerød, Denmark) in transmittance mode to simultaneously determine contents of moisture, fat, and protein. The 180 g sample of ground, homogenized meat at 20 °C was placed into FoodScan™ cups with a diameter of 140 mm and a height of 17.5 mm. The spectrum of each sample in the near-infrared range (850–1050 nm in 2 nm increments) was then determined from 20 measurements. The result provided by FoodScan™ was presented as the reciprocal of transmittance [log (1/T)].

### 2.5. Fatty Acids Profile

Fatty acid profile analysis was performed as follows: a volume of 3 mL of 2 M NaOH was added to 50 mg (±3 mg) of the homogenized sample, placed in an incubator for 40 min at 90 °C, and stirred every 10 min. A volume of 2 mL of diethyl ether was used to extract the samples. Extraction was performed three times. Derivatization was performed with boron trifluoride. The separation and quantification of the fatty acid methyl esters (FAME) were performed using a Bruker 456-GC gas chromatograph equipped with an FID flame ionization detector and a CP 7420 capillary column 100 m long, 0.25 mm in diameter, and 0.25 μm porosity. Hydrogen was used as carrier gas at a constant flow rate of 1.3 mL/min. The chromatographic conditions were as follows: the injector temperature was 200 °C, and the detector temperature was 250 °C. The oven temperature of the chromatograph was kept at 120 °C for the first 7 min, then it increased at a rate of 7 °C/min to 140 °C. After 10 min, the temperature increased from 140 °C to 240 °C at a rate of 4 °C/min. The results are presented as peaks. Individual FAME was identified by comparing their retention times with those of validated standards (37 FAME Mix, Sigma Aldrich, Bellefonte, PA, USA) and results are expressed as a percentage of total FAMEs.

The total contents of saturated fatty acids (SFA), monounsaturated fatty acids (MUFA), polyunsaturated fatty acids (PUFA), PUFA n-6, PUFA n-3, and PUFA n-6/n-3 were calculated. The method of Medeiros et al. [20] was used to calculate the desirable hypocholesterolemic fatty acids (DFA):

(1) DFA=MUFA+PUFA+C18:0

and hypercholesterolemic fatty acids (OFA)

(2) OFA=C12:0+C14:0+C16:0

Atherosclerotic (AI) and thrombogenic (TI) indices [21] were also calculated according to the following equations:

(3) $$AI=\frac{\;(C12:0+4\times C14:0+C16:0)}{MUFA+\Sigma PUFA(n-6)+\Sigma PUFA(n-3)}$$

(4) $$TI=\frac{\;C14:0+C16:0+C18:0}{0.5\times MUFA+0.5\times (n-6)+3\times (n-3)+\frac{\;n-3}{n-6}}$$

The ratio of hypocholesterolemia to hypercholesterolemia (h/H) was calculated according to Ivanova and Hadzhinikolova [22]:

(5) $$\frac{h}{H\;}=\frac{\;C18:1n-9+C18:2N-6+C18:3n-3}{C12:0+C14:0+C16:0}$$

### 2.6. pH Value

The pH value was measured according to ISO 2917:1999. To measure the pH, a 10 g minced sample was homogenized with 10 times the weight of the potassium chloride solution for 1 min using a homogenizer (IKA ULTRA-TURRAX T25 Basic, Staufen, Germany). The pH was measured using a digital pH meter CPC-501 (Elmetron, Zabrze, Poland) equipped with a pH electrode (ERH-111, Hydromet s.c., Gliwice, Poland) with temperature compensation.

### 2.7. Water Activity

Portions of meat without fat were separated for sample preparation. The water activity of minced meat was measured triplicate in samples of 10 g at 20 °C in the Lab-master a_w_ instrument (Novasina AG, Lachen, Switzerland). The apparatus was calibrated using standard salts in the a_w_ range of 0.7 to 1.

### 2.8. Lipid Oxidation

Lipid oxidation was measured by the TBARS test. Minced sample (3 g) has been homogenized with mixture of 12 mL cold trichloroacetic acid (TCA 4%) (POCH S.A., Gliwice, Poland) with 0.2 mL of alcoholic butylated hydroxytoluene (BHT 0.01%) (Sigma-Aldrich Chemie GmbH, Steinheim am Albuch, Germany). Next, samples were shaken, filtered, and heated at 100 °C for 20 min after the 0.65 mL 2-thiobarbituric acid (TBA) (POCH S.A., Gliwice, Poland) addition. The TBARS content was measured using U-5100 UV-VIS spectrophotometer (HITACHI High America Inc., Dallas, TX, USA) after cooling and centrifugation. The TBARS value was estimated using the following equation:

(6) $$TBARS=5.5\times A_{532}$$

where A_532_ refers to the absorbance measured at 532 nm. The result was expressed as mg MDA per 1 kg sample.

### 2.9. Color Parameters

The CIE LAB system was used to determine the parameters of the color of the freshly minced meat by the reflection method using an 8200 Series (X-Rite Inc., Grand Rapids, MI, USA) spherical spectrophotometer with D65 illuminant and 10° standard observer and a 12 mm port/viewing area. About 20 g of minced sample was placed in a thin, transparent cling film. Then, the sample was placed to completely cover the measuring hole. The results were expressed as lightness (L*, 100 = white and 0 = black), redness (a*), and yellowness (b*). Due to the heterogeneity of the sample, ten measurements were taken at random different locations on the surface of sample.

### 2.10. Microbial Analysis

Microbiological tests to determine total viable count (TVC) were performed according to ISO 4833-2:2013 [23]. Lactic acid bacteria (LAB) were determined according to ISO 15214:1998 [18]. *Enterobacteriaceae* was determined according to ISO 21528-2:2017 [24]. The population of *Escherichia coli* was determined according to ISO 16649-1:2018 [25]. *Clostridium perfringens* were determined according to ISO 7937:2004 [26]. The concentration of colony-forming units is reported using logarithmic notation (log CFU g^−1^). *Listeria monocytogenes* was determined according to ISO 11290-1:2017 [27]. In this case, the presence of 25 g of the sample was tested.

### 2.11. Statistical Analysis

The Statistica v. 13 software (TIBCO Software Inc., Palo Alto, CA, USA) was used to perform the statistical analysis of the data from the experiment. Data were analyzed using a multiway analysis of variance (MANOVA) preceded by the verification of primary assumptions. The post hoc test was performed with Tukey test at α = 0.05. Baseline statistics are presented as mean ± standard deviation.

## 3. Results and Discussion

### 3.1. Chemical Properties of Lamb

Meat from Wrzosówka lambs analyzed at the beginning of the experiment contained 75% water, 18% protein, slightly more than 7% fat, 2.3% collagen, and 0.28% sodium (Table 2). This chemical composition is similar to results published by other authors [28], although fluctuations caused by breeding conditions, among others, are possible. In the lamb meat studied, mainly SFA and MUFA were found (Table 2). The meat of small ruminants is characterized by a significant content of saturated fatty acids since PUFAs from the diet are subject to a biohydrogenation process carried out by rumen microorganisms, making the fatty acid (FA) profile of the meat different from that in the diet [29]. Palmitic acid (C:16), stearic acid (C18:0), and oleic acid (C18:1) accounted for the largest share of FA. Oleic acid was the most abundant for unsaturated fatty acids and all detected acids. This frequency of occurrence is consistent with the generally accepted values for fatty acids in lamb meat [30]. However, lamb meat has a low n-6/n-3 ratio compared to other meats, such as pork and chicken (3.7 vs. 6.4 and 5.0, respectively) [31]. It is also an interesting source of conjugated linoleic acid (CLA), particularly the cis-9, trans-11 isomer (also called rumenic acid), as well as α-linolenic acid (C18:3 n-3) and its elongation products [32]. Other lipid quality indicators also indicate high nutritional value. The content of desirable DFA, which is the sum of unsaturated fatty acids and stearic acid (C18:0), was 5.46. The content of OFA, which is influenced by the content of saturated fatty acids such as lauric acid (C12:0), myristic acid (C14:0), and palmitic acid (C16:0), was 1.7. The nutritional indices AI, TI, and h/H are calculated to assess the nutritional quality of the fat composition of meat. TI and AI indices express the potential impact of individual fatty acids on human health and are primarily associated with the risk of developing cardiovascular diseases. AI indicates the risk of fat deposition in the walls of the arteries, causing atherosclerosis, while TI determines the possibility of blood clots [21]. The higher the values of these coefficients, the greater the risk of developing cardiovascular disease [20], and their value should not exceed 1.0 [33]. Our research shows that Wrzosówka lamb meat has a high protective potential against cardiovascular health problems. The h/H ratio is used to express the functional effect of fatty acids on cholesterol metabolism. It is related to the functional activity of fatty acids in lipoprotein metabolism for plasma cholesterol transport and the risk of cardiovascular disease. A relatively high h/H ratio and low AI and TI values reduce the incidence of cardiovascular disease [34].

Acidity, lipid oxidation, water activity, and color parameter values of fresh lamb meat are shown in Table 3. The pH24 is used to assess the shelf life and quality of meat and its suitability for processing. The normal pH drop is from 7.0–7.2 to 5.5–5.7 in about 24 h. Meat with a final pH of about 5.5–5.8 has the most desirable quality characteristics and the highest tenderness [35]. The pH24 of the meat was at about 5.6. The final pH values are, therefore, within the range recommended for high-quality meat. Similar pH values were obtained by other researchers [30,35,36] using lamb meat in their studies.

High water activity promotes the hydrolysis of fats and the degradation of proteins, causing changes in the color, aroma, and taste of meat [37]. It is also particularly susceptible to microbial growth even during cold storage [4]. The water activity of the lamb meat tested was 0.972 (Table 3). This demonstrates the naturally high susceptibility of fresh meat to microbial spoilage. Similar results for lamb meat were obtained by Karwowska et al. [38]

Lipid peroxidation in red meat is determined by the concentration of thiobarbituric acid reactive substances (TBARS), mainly malonaldehyde (MDA) [39]. Malonaldehyde is one of the most abundant aldehydes formed during secondary lipid oxidation, and probably the most widely used as an oxidation marker. It is a reactive aldehyde that forms interactions with nucleic acids and covalent protein adducts, thus contributing to its toxicity, especially in meat and meat products. It is usually considered a biomarker of oxidative stress in the body. The fatty acid profile of red meat affects TBARS concentrations, especially the susceptibility of unsaturated fatty acids to oxidation [40]. Ripol et al. [41] considered the threshold for detecting rancidity and off-flavors by trained sensory panelists and proposed the acceptability level of MDA of 1 mg per 1 kg of lamb meat. The initial content of malonaldehyde in lamb meat was well below this value and was 0.62 mg (Table 3).

Meat color is one of the most important traits used by consumers as a visual indicator of freshness quality and consumer acceptance of meat. Chromatic component (a*) along with brightness (L*) are the main characteristics of lamb meat color [36], while the chromatic component (b*) is considered in science and the meat industry as an indicator of fat color. Consumers will characterize red meat as high quality when its lean tissue has a bright, cherry red color [42]. From an analytical standpoint, this means that fresh lamb meat should have values higher than 9.5 for a* and 34 for L* to be considered acceptable by consumers, and these values should rise to at least 14.5 and 44 for a* and L*, respectively, to satisfy 95% of consumers. Wrzosówka lamb meat was characterized by a redness level of more than 23 and a brightness level of 52 (Table 3).

### 3.2. Effect of Acid Whey Addition and Ultrasonic Treatment on Chemical Stability of Lamb Stuffing

Table 4 shows the results of pH, water activity, secondary fatty acid oxidation products (TBARS), and color parameters (CIELAB) of meat stuffing. The addition of salt or curing salt (99.6% salt and 0.04% nitrite) caused a significant increase in the pH value of the freshly prepared stuffing (*p* < 0.05). Sodium chloride is an essential multifunctional ingredient in meat products, which is added to improve organoleptic properties, microbiological safety, and technological functionality, such as water retention capacity. Chloride ions bind to myofibrillar proteins, increasing their net negative charge [43], thereby increasing the amount of sodium cations in the environment and causing an increase in pH. The increase in pH promotes microbial growth, but the addition of salt, by reducing water activity and the action of chloride ions, at the same time can effectively inhibit microbial growth in the meat product [44]. The addition of acid whey alone (W, UW) caused a significant (*p* < 0.05) reduction in the pH of the fresh stuffing. This was as expected since the pH of the whey used was 4.15. At the same time, it was found that meat sonication did not significantly modify the pH value of fresh meat stuffing. According to Al-Khalasi and Mahgoub [45], the desired pH for lamb meat is in the range of 5.4 to 5.8, close to the values established for fresh stuffing on day 0 of the experiment. After 5 days of storage, the pH of the control sample was found to increase. Similarly, many authors observed an increase in pH during the storage of shredded meat [46]. The increase in meat pH may be due to the release of microbial metabolites and/or endogenous enzymes, which accelerate meat spoilage by hydrolyzing proteins and forming volatile compounds (ammonia and trimethylamine) [47]. Meat exhibiting an elevated pH value (6.0–6.4) spoils faster than more acidic meat with a pH in the range of 5.3–5.7. A positive correlation is found between the average values of the total bacterial count and the pH value in minced meat [48]. Meanwhile, after 5 days of storage, a significant (*p* < 0.05) reduction in pH values was observed in samples with salt (S, US), whey (W, UW), and salt × whey (SW, USW). The greatest reduction was observed in the sample with the addition of whey alone. The decrease in the pH value of samples with the addition of acid whey can be explained by the acidity of the whey added to the samples, and/or the dissociation of lactic acid. Studies by other authors [46] confirm that the addition of natural antioxidants, such as lactic acid from whey, can also lower the pH of meat products during storage. In addition, as in other studies [49], we observed an increase (*p* < 0.05) in the number of all microorganisms analyzed during storage, especially LAB, representing the predominant bacteria that cause meat spoilage. Therefore, the lower pH value observed in our results after 5 days of storage may be due to the acidification of the medium caused by an increase in the number of lactic acid bacteria, which usually occurs when meat is vacuum-packed [49]. Sonication of the stuffing did not cause significant changes in pH values. Although a change in the acidity of ripened meat after sonication is observed [11], the effect is weak in the case of stuffing due to the limited impact (sample area) and is compensated for, among others, by organic compounds released from cells.

Stuffing showed high water activity, affecting product perishability and demonstrating the need for food preservation techniques (Table 4). In order to reduce microorganisms in meat and ensure food quality, an integrated approach (simultaneous application of several factors) is recommended [50]. Even though a set of factors acting on microorganisms (salt, sodium nitrite, whey, and ultrasound) was applied, all samples, after 5 days of storage, showed a_w_ of 0.963–0.973. The sonication of stuffing does not significantly change the value of water activity, thereby not significantly changing the microbiological susceptibility of stuffing.

The oxidative stability of the stuffing after 5 days of storage was evaluated (Table 4). A low content of malonaldehyde was found, not exceeding 1 mg per kilogram of stuffing in all variants. Samples containing sodium nitrite (C, UC) had the lowest content of secondary fat oxidation products reacting with thiobarbituric acid, especially on day 0. However, samples without sodium nitrite (K, UK) and samples with whey (W, UW) were similar. The antioxidant properties and mechanisms of action of sodium nitrite are generally known. Other authors confirm that the addition of nitrate has an effect on lowering TBARS [38]. As stated by Honikel [51], nitrates act against lipid oxidation mainly due to oxygen deletion. The nitric oxide formed from nitrates can be oxidized to form NO_2_, causing oxygen sequestration. Oxidation of meat lipids is inhibited under such conditions. Studies by other researchers [52] demonstrated that acid whey can replace nitrites in terms of antioxidant effects. The antioxidant effect of whey proteins is mainly associated with beta-lactoglobulin and lactoferrin due to their ability to inactivate pro-oxidant heme proteins (ferrillioglobin) and bind iron [53]. Thus, the use of whey for minced lamb meat seems justified by the fact that lamb meat contains a high amount of heme iron, making it susceptible to oxidative changes. Our study does not fully confirm the antioxidant effect of whey. We observed a significant (*p* < 0.05) increase in MDA in samples containing whey alone (W, UW). In this situation, it can be assumed that the addition of acid whey resulted in the introduction of additional fatty acids into the stuffing, including CLA isomers [52], which were oxidized during the storage process, increasing the content of secondary fat oxidation products. Similarly, significantly (*p* < 0.05) higher MDA content was found in samples containing salt (S, US and SW, USW). Sodium chloride (NaCl) can act as a prooxidant in meat and meat products [54]. However, the pro-oxidant effect of NaCl depends on its concentration. The antioxidant effect of low doses of salt (up to 2%) can be explained by the effect of sodium chloride on reducing water activity, which delays fat oxidation [55]. In contrast, higher concentrations of NaCl promote the release of iron ions from heme proteins in muscle, which in turn initiates lipid autoxidation and inhibits the activity of antioxidant enzymes [56]. Unfortunately, the combined effects of whey and salt did not confirm their synergistic antioxidant effects. The SW and USW samples showed significantly higher MDA content but were still at an acceptable level (<1 mg). Interestingly, the use of ultrasound caused the MDA content to decrease significantly (*p* < 0.05) in the samples with added salts (US, USW) after 5 days of storage. As a result of ultrasound, salts diffuse faster, increasing the effectiveness of the salt’s effect on the fats in the stuffing.

The color of meat products is an important parameter determining consumer acceptance. It is very labile, as it depends on a combination of many different factors, such as fat content, moisture content, chemical form, myoglobin concentration, and physical structure. The CIELAB values of meat stuffing samples are shown in Table 4. It was found that the brightness of the color (L*) and its chromatic component (b*) for all stuffing variants were at a similar level, ranging from 47 to 53 and 9 to 14, respectively. In the case of the chromatic component (a*), the stuffing described values ranging from 8 to 10, except for samples with added nitrite (C and UC). At the same time, fluctuations were found in the values of all color attributes in all variants. Contrary to expectations, the addition of acid whey did not increase the brightness and redness of the stuffing, as indicated by the results of other authors [57]. The a* value was significantly higher in the sample with the addition of nitrite, which was expected since nitrite contributes to the formation of nitrosyl myoglobin in meat products. Lower, statistically insignificant (*p* < 0.05) values of the b* parameter were observed in the samples with added salt (S, SW, US and USW). This could have been due to reduced oxygen solubility trapped by the salt concentration [58], as well as anaerobic conditions that promoted the formation of brownish metmyoglobin [57]. Sonication of the stuffing did not modify the parameters of its color.

### 3.3. Effect of Acid Whey Addition and Ultrasonic Treatment on Microbiological Stability of Lamb Stuffing

Lamb meat, especially minced meat, is a good medium for the growth of microorganisms both saprophytic (spoilage bacteria), causing spoilage, and pathogenic ones. This is due to the natural chemical composition and high level of water activity. The first group of bacteria that significantly contribute to meat deterioration include *Enterobacteriaceae* and lactic acid bacteria (LAB) [59,60]. Although the latter consists of a very diverse group of microorganisms that can cause food spoilage or, on the contrary, protect against spoilage and pathogen development [61]. Saprophytic microorganisms usually do not affect safety but can determine changes in the physical and chemical properties of meat. Sheep meat can be contaminated with pathogens, such as *Escherichia coli*, *Listeria monocytogenes*, and *Clostridium perfringens* [3,60], which degrade food safety because they can pose a health risk to consumers. The contents of TVC, LAB, *Enterobacteriaceae*, *E. coli*, *L. monocytogenes*, and *Cl. perfringens*, in fresh samples (0 days) and samples after 5 days of vacuum storage in the cold room (+4 °C) are shown in Table 5.

TVC in fresh minced lamb meat (K) was at 5.10 log CFU g^−1^ (Table 5). Curing and salting did not affect the TVC value. The addition of liquid acid whey caused a significant (*p* < 0.05) increase in TVC to a level of 5.60 in S and SW samples. Sonication caused a non-significant (*p* < 0.05) decrease in TVC values in all fresh samples (0 days). In the control (K), which was stored in the cold (+4 °C) vacuum for 5 days, there was a significant (*p* < 0.05) increase in TVC, both in the non-sonicated and sonicated samples, of 0.73 and 0.84 log units, respectively. A significant (*p* < 0.05) increase in TVC, by about 0.6 logarithmic units, was also observed in the whey (W, UW) and whey-salted (SW, USW) samples. At the same time, the TVC value in these samples was higher (*p* < 0.05) than in the control. Raw minced meat products are highly susceptible to chemical and microbial spoilage due to their large interfacial area. Meat processing involving operations of mincing, mixing, etc., promotes contamination of the material and the growth of microorganisms, as these operations develop the surface of the stuffing, increasing susceptibility to spoilage. However, TVC values in the samples were not found to exceed 7 log CFU g^−1^, which is considered the limit of microbiological acceptability for mutton meat [46,49]. Nitrite was the most effective preservative and significantly (*p* < 0.05) decreased TVC after 5 days of storage, which is consistent with the results of other authors [62]. Nitrite has a high antimicrobial activity due to the reduction of microbial oxygen uptake and metabolic enzymes and alters the electron transport chain [40]. Additionally, the addition of salt reduced the TVC value (day 5). In contrast, the sonication of the stuffing had no effect on the TVC value of the stored samples. The microbiological effects of stuffing sonication are virtually limited to the surface in contact with the transducer, where ultrasonic cavitation occurs. The physical structure of the stuffing favors the absorption of the waves and makes their energy content over a short distance fall below the cavitation threshold, practically excluding the effect of microbial destruction.

The LAB are facultative anaerobic bacteria and are a significant part of the meat microflora because they can grow at low oxygen concentrations [63]. The LAB content of the fresh control sample (K) was found to be 6.30 log CFU g^−1^. The addition of nitrite/salt reduced the LAB count to 5.73 and 5.90 log CFU g^−1^, respectively (Table 5). Since acid whey from unpasteurized milk is a source of lactic acid bacteria, its addition to the stuffing (W, SW, UW, and UWS) increased (*p* < 0.05) the number of LAB, on average, by 1 log CFU, compared to samples without this addition. Ultrasound had no significant effect on LAB values in fresh samples (0 days). After 5 days of refrigerated storage in a vacuum, there was a significant (*p* < 0.05) increase in the number of LAB in all samples. Nitrite (C, UC) and salt (S, US) did not inhibit LAB growth. As reported by other authors [64], lactic acid bacteria are quite insensitive to nitrite and contained higher levels of LAB in products. Sonication of meat stuffing was not shown to have a significant effect on the number of LAB in stored samples.

As for *Enterobacteriaceae*, which, along with *E. coli*, are usually considered an indicator of hygiene, their content on day 0 ranged from 2.48 log CFU g^−1^ to 2.74 log CFU g^−1^ (Table 5), indicating good quality minced lamb meat. The addition of acid whey and sonication had no effect (*p* < 0.05) on the *Enterobacteriaceae* value of fresh samples (day 0). After 5 days of storage, significant differences were noted in the level of *Enterobacteriaceae* contamination of the stuffing. Sonication, and the addition of nitrite, whey, and salt significantly reduced the value of these bacteria: (UC, USW, UW) < (US, W, SW, C) < (UK, K). As reported by Chen et al. [65], the competitive nature of lactic acid bacteria in meat products with lowered pH and high NaCl concentration is responsible for the reduced survival rate of *Enterobacteriaceae*. Lowering the pH may partially explain the reduction and disappearance of these bacterial groups, as observed by other authors [52].

*E. coli* is used as an indicator of fecal coliform contamination, and according to Commission Regulation (EC) No 1441/2007 [66], their content in raw minced meat should not be higher than 2.7 log CFU g^−1^ (Table 5). The fresh samples (day 0) were of satisfactory quality (*E. coli* < 1.7 CFU g^−1^). After 5 days, there was a significant (*p* < 0.05) increase in *E. coli* in all samples. No treatment was found to affect the level of contamination with these bacteria. Nitrite added to the stuffing effectively inhibited *L. monocytogenes* and *Cl. perfringens*, as confirmed by other authors [7].

## 4. Conclusions

The results show that ultrasonic treatment (low frequency and medium intensity) of fresh lamb meat stuffing with the addition of acid whey increases its safety and shelf life in terms of its use in meat products. The greatest modification of the stuffing properties is caused by the addition of whey. The addition of salt and whey lowers the pH value of meat stuffing during storage. All variants of stuffing were characterized by a high level of oxidative stability. After 5 days of stuffing storage, the content of malonaldehyde was below 1 mg per 1 kg of stuffing. The addition of salt and whey to the stuffing reduced the formation of chemical degradation products of fat. The combinations of additives (salt, curing salt, and whey) applied to the stuffing did not modify its water activity or color parameters. There was no significant effect of ultrasonic treatment of stuffing on chemical properties of technological importance. Stuffing sonication did not modify the number of LAB but significantly reduced the number of *Enterobacteriace*. The addition of whey and salt enhanced the effect of sonication, and the result was comparable to samples with the addition of nitrates. Treatment of raw stuffing from fresh lamb meat with acid whey and salt for 2.5 min with low-frequency, medium-intensity ultrasound is an alternative to the use of nitrates due to the oxidative and microbiological stability of the stuffing.

## Figures and Tables

**Table 1:** Ingredients and processing of lamb stuffing.

Variant	Brine (99.5% Salt, 0.5% Sodium Nitrite) [%]	Salt [%]	Water [%]	Liquid Acid Whey [%]	Sonication [Yes/No]
K	-	-	-	-	No
C	2	-	10	-	No
S	-	2	10	-	No
W	-	-	-	10	No
SW	-	2	-	10	No
UK	-	-	-	-	Yes
UC	2	-	10	-	Yes
US	-	2	10	-	Yes
UW	-	-	-	10	Yes
USW	-	2	-	10	Yes

**Table 2:** Proximate and fatty acid composition of lamb (mean ± standard deviation).

Proximate Composition of Meat		
	Moisture (%)	74.9 ± 3.80
	Fat (%)	7.36 ± 0.82
	Proteins (%)	18.1 ± 0.50
	Collagen (%)	2.30
	Sodium (%)	0.28
Fatty acid composition (more than 0.05% FAME)		
SFA	C10:0	0.23 ± 0.02
	C12:0	0.18 ± 0.01
	C14:0	2.36 ± 0.23
	C15:0	0.61 ± 0.02
	C16:0	20.47 ± 0.32
	C17:0	1.40 ± 0.01
	C18:0	24.08 ± 0.25
	C20:0	0.23 ± 0.02
MUFA	C14:ln5	0.12 ± 0.01
	C16:ln7	1.77 ± 0.51
	C17:ln7	0.81 ± 0.02
	C18:ln9c + C18:ln9t	43.65 ± 1.65
	C20:ln9	0.11 ± 0.01
PUFA	C18:2n6c	2.28 ± 0.32
	C18:3n3(alpha)	0.66 ± 0.04
	C20:2n6	0.11 ± 0.03
	C20:4n6	0.18 ± 0.01
	C20:5n3	0.07 ± 0.02
The nutritional indices of fat		
	SFA (%)	49.77
	MUFA (%)	46.67
	PUFA (%)	3.56
	PUFA n-3 (%)	0.72
	PUFA n-6 (%)	2.64
	PUFA n-9 (%)	43.76
	PUFA n-6/n-3	3.66
	DFA	5.46
	OFA	1.70
	AI	0.60
	TI	0.78
	h/H	2.02

SFA—saturated fatty acids; MUFA—monounsaturated fatty acids; PUFA—polyunsaturated fatty acids; DFA—hypocholesterolemic fatty acids; OFA—hypercholesterolemic fatty acids; AI—atherogenic indices; TI—thrombogenic indices; h/H—hypocholesterolemia to hypercholesterolemia ratio.

**Table 3:** Acidity, lipid oxidation, water activity, and color parameters of lamb (mean ± standard deviation).

Parameter	Value
Acidity (pH24)	5.56 ± 0.06
Water activity	0.972 ± 0.003
Lipid oxidation (mg malonaldehyde kg^−1^)	0.62 ± 0.10
CIE Lightness L*	52.27 ± 2.02
CIE Redness a*	23.68 ± 0.37
CIE Yellowness b*	16.60 ± 1.46

**Table 4:** Acidity, lipid oxidation, water activity, and color parameters of lamb stuffing (mean ± standard deviation) before and after 5-day storage (mean ± standard deviation).

	Day	K	C	S	W	SW	UK	UC	US	UW	USW
pH	0	5.62 ^Aa^ ± 0.04	5.72 ^Ba^ ± 0.01	5.71 ^Ba^ ± 0.04	5.56 ^Ca^ ± 0.01	5.63 ^Aa^ ± 0.04	5.68 ^Aa^ ± 0.02	5.73 ^Ba^ ± 0.03	5.66 ^Ba^ ± 0.02	5.55 ^Ca^ ± 0.00	5.65 ^Ba^ ± 0.01
	5	5.83 ^Eb^ ± 0.01	5.65 ^Ba^ ± 0.03	5.32 ^Cb^ ± 0.02	5.01 ^Db^ ± 0.02	5.34 ^Cb^ ± 0.01	5.79 ^Eb^ ± 0.02	5.72 ^Ba^ ± 0.05	5.37 ^Cb^ ± 0.01	5.16 ^Ab^ ± 0.04	5.38 ^Cb^ ± 0.04
a_w_	0	0.971 ^Aa^ ± 0.02	0.970 ^Aa^ ± 0.04	0.970 ^Aa^ ± 0.03	0.972 ^Aa^ ± 0.01	0.971 ^Aa^ ± 0.01	0.973 ^Aa^ ± 0.01	0.972 ^Aa^ ± 0.03	0.973 ^Aa^ ± 0.02	0.975 ^Aa^ ± 0.04	0.974 ^Aa^ ± 0.03
	5	0.965 ^Ab^ ± 0.01	0.963 ^Ab^ ± 0.02	0.964 ^Aa^ ± 0.02	0.963 ^Ab^ ± 0.03	0.970 ^Ba^ ± 0.03	0.967 ^Ab^ ± 0.03	0.965 ^Ab^ ± 0.02	0.966 ^Aa^ ± 0.01	0.969 ^Aa^ ± 0.02	0.973 ^Ba^ ± 0.01
TBARS (mg MDA kg^−1^)	0	0.24 ^Aa^ ± 0.13	0.13 ^Aa^ ± 0.00	0.40 ^Ba^ ± 0.08	0.27 ^Aa^ ± 0.03	0.57 ^Ca^ ± 0.21	0.17 ^Aa^ ± 0.02	0.24 ^Aa^ ± 0.00	0.44 ^Ba^ ± 0.00	0.26 ^Aa^ ± 0.02	0.52 ^Ca^ ± 0.08
	5	0.31 ^Aa^ ± 0.02	0.27 ^Ab^ ± 0.04	0.35 ^Aa^ ± 0.01	0.39 ^Ab^ ± 0.04	0.43 ^Ab^ ± 0.01	0.28 ^Ab^ ± 0.04	0.29 ^Aa^ ± 0.04	0.31 ^Ab^ ± 0.04	0.33 ^Ab^ ± 0.02	0.39 ^Ab^ ± 0.03
L*	0	50.41 ^Aa^ ± 4.69	50.41 ^Aa^ ± 6.49	47.90 ^Aa^ ± 3.25	51.17 ^Aa^ ± 2.82	48.89 ^Aa^ ± 3.67	50.64 ^Aa^ ± 2.81	46.63 ^Aa^ ± 3.70	48.65 ^Aa^ ± 4.61	52.38 ^Aa^ ± 3.11	48.73 ^Aa^ ± 3.84
	5	52.16 ^Aa^ ± 3.32	48.68 ^Aa^ ± 3.81	48.40 ^Aa^ ± 2.49	53.19 ^Aa^ ± 2.25	51.14 ^Aa^ ± 4.72	52.91 ^Aa^ ± 2.53	50.64 ^Aa^ ± 2.81	49.09 ^Aa^ ± 2.61	53.92 ^Aa^ ± 2.53	49.24 ^Aa^ ± 3.06
a*	0	9.05 ^Aa^ ± 1.68	14.04 ^Ba^ ± 3.43	8.39 ^Aa^ ± 2.24	8.33 ^Aa^ ± 1.28	7.70 ^Aa^ ± 1.33	8.34 ^Aa^ ± 1.32	15.56 ^Ba^ ± 1.89	8.41 ^Aa^ ± 1.58	7.92 ^Aa^ ± 1.40	8.38 ^Aa^ ± 1.54
	5	9.07 ^Aa^ ± 1.46	16.39 ^Ba^ ± 2.06	9.46 ^Aa^ ± 1.10	8.45 ^Aa^ ± 0.93	8.13 ^Aa^ ± 1.85	8.41 ^Aa^ ± 1.09	14.34 ^Aa^ ± 1.32	10.17 ^Aa^ ± 2.15	8.45 ^Aa^ ± 1.37	8.11 ^Aa^ ± 1.06
b*	0	11.44 ^Aa^ ± 1.22	11.73 ^Aa^ ± 3.35	10.24 ^Aa^ ± 0.75	11.79 ^Aa^ ± 0.93	9.81 ^Aa^ ± 1.29	11.60 ^Aa^ ± 0.99	12.11 ^Aa^ ± 1.09	10.68 ^Aa^ ± 1.69	12.0 ^Aa^ ± 0.84	10.51 ^Aa^ ± 1.21
	5	11.80 ^Aa^ ± 1.26	13.54 ^Aa^ ± 1.01	10.70 ^Aa^ ± 1.12	11.95 ^Aa^ ± 0.61	11.10 ^Aa^ ± 1.23	11.85 ^Aa^ ± 1.04	11.60 ^Aa^ ± 0.99	11.39 ^Aa^ ± 1.04	12.30 ^Aa^ ± 1.19	10.18 ^Aa^ ± 1.11

Sample: K—control; C—cured; S—salted; W—with acid whey; SW—with salt and acid whey; UK—sonicated control; UC—sonicated cured; US—sonicated salted; UW—sonicated with acid whey; USW—sonicated salted with acid whey; MDA—malonaldehyde. Homogenous group in the column are marked with the same lowercase letters ^a,b^. Homogenous group in the row marked with the same capital letter ^A–D^.

**Table 5:** Microbial counts (log CFU g^−1^) of lamb stuffing before and after 5-day storage (mean ± standard deviation).

Bacteria	Day	K	C	S	W	SW	UK	UC	US	UW	USW
LAB	0	6.30 ^Ba^ ± 0.12	5.73 ^Aa^ ± 0.15	5.90 ^Aa^ ± 0.15	7.30 ^Ca^ ± 0.17	7.30 ^Ca^ ± 0.15	6.28 ^Ba^ ± 0.15	5.70 ^Aa^ ± 0.15	5.86 ^Aa^ ± 0.16	7.27 ^Ca^ ± 0.16	7.26 ^Ca^ ± 0.16
	5	7.80 ^Bb^ ± 0.15	7.03 ^Ab^ ± 0.15	7.15 ^Ab^ ± 0.15	8.10 ^Bb^ ± 0.17	8.00 ^Bb^ ± 0.14	7.86 ^Bb^ ± 0.15	7.15 ^Ab^ ± 0.15	7.18 ^Ab^ ± 0.14	8.16 ^Bb^ ± 0.16	8.11 ^Bb^ ± 0.15
*Cl. perfringens*	0	<10									
	5										
*Enterobacteriaceae*	0	2.74 ^Aa^ ± 0.08	2.66 ^Aa^ ± 0.08	2.73 ^Aa^ ± 0.09	2.52 ^Aa^ ± 0.08	2.62 ^Aa^ ± 0.07	2.71 ^Aa^ ± 0.08	2.62 ^Aa^ ± 0.08	2.70 ^Aa^ ± 0.08	2.48 ^Aa^ ± 0.08	2.57 ^Aa^ ± 0.07
	5	3.53 ^Db^ ± 0.08	2.30 ^Bb^ ± 0.15	2.66 ^Ca^ ± 0.08	2.26 ^Bb^ ± 0.08	2.28 ^Bb^ ± 0.09	3.39 ^Db^ ± 0.07	2.01 ^Ab^ ± 0.08	2.45 ^Bb^ ± 0.07	2.10 ^Ab^ ± 0.04	2.03 ^Ab^ ± 0.07
*E. coli*	0	1.68 ^Ba^ ± 0.11	1.48 ^Aa^ ± 0.20	1.56 ^Ba^ ± 0.13	1.43 ^Aa^ ± 0.12	1.64 ^Ba^ ± 0.10	1.64 ^a^ ± 0.10	1.43 ^Aa^ ± 0.18	1.52 ^Aa^ ± 0.11	1.39 ^Aa^ ± 0.09	1.61 ^Ba^ ± 0.09
	5	2.15 ^Ab^ ± 0.17	1.85 ^Ab^ ± 0.14	2.00 ^Ab^ ± 0.16	2.18 ^Ab^ ± 0.13	2.02 ^Ab^ ± 0.12	2.11 ^Ab^ ± 0.15	1.80 ^Ab^ ± 0.12	1.98 ^Ab^ ± 0.13	2.09 ^Ab^ ± 0.11	1.98 ^Ab^ ± 0.10
*L. monocytogenes*	0	not present									
	5										
TVC	0	5.10 ^Aa^ ± 0.15	5.08 ^Aa^ ± 0.19	5.11 ^Aa^ ± 0.16	5.60 ^Ba^ ± 0.14	5.62 ^Ba^ ± 0.15	5.08 ^Aa^ ± 0.14	5.05 ^Aa^ ± 0.17	5.08 ^Aa^ ± 0.14	5.57 ^Ba^ ± 0.13	5.58 ^Ba^ ± 0.14
	5	5.83 ^Bb^ ± 0.12	4.74 ^Aa^ ± 0.17	4.88 ^Aa^ ± 0.12	6.22 ^Bb^ ± 0.11	6.15 ^Bb^ ± 0.14	5.92 ^Bb^ ± 0.13	4.79 ^Aa^ ± 0.19	4.91 ^Aa^ ± 0.15	6.28 ^Bb^ ± 0.13	6.19 ^Bb^ ± 0.16

Sample: K—control; C—cured; S—salted; W—with acid whey; SW—with salt and acid whey; UK—sonicated control; UC—sonicated cured; US—sonicated salted; UW—sonicated with acid whey; USW—sonicated salted with acid whey. Homogenous group in the column are marked with the same lowercase letters ^a,b^. Homogenous group in the row marked with the same capital letter ^A–D^.

## Data Availability

Data is contained within the article.
